# Clinical correlation of anti-desmoglein antibody dynamics in pemphigus treated with rituximab

**DOI:** 10.3389/fimmu.2025.1713987

**Published:** 2025-12-12

**Authors:** Vishvan Naidu, Harshita A. Jain, Ali Yalcinkaya, Yathavi Charavanamuttu, Dhruva Biswas, Nadira Ali, John B. Mee, Jitesh Chauhan, Kristina Semkova, Emma C. Benton, Sophia N. Karagiannis, Jane Setterfield, Richard W. Groves, Thomas J. Tull

**Affiliations:** 1 St. John’s Institute of Dermatology, School of Basic & Medical Biosciences & KHP Centre for Translational Medicine, King’s College London, London, United Kingdom; 2 Department of Cardiovascular Medicine, Yale School of Medicine, New Haven, CT, United States; 3 Immunodermatology Laboratory, Synnovis Analytics, St Thomas’ Hospital, London, United Kingdom; 4 St John’s Institute of Dermatology, Guy’s and St Thomas’ NHS Foundation Trust, London, United Kingdom; 5 Breast Cancer Now Research Unit, School of Cancer & Pharmaceutical Sciences, King’s College London, Innovation Hub, Guy’s Hospital, London, United Kingdom; 6 Centre for Host Microbiome Interactions (CHMI), Faculty of Dentistry, Oral & Craniofacial Sciences, King’s College London, London, United Kingdom

**Keywords:** pemphigus, rituximab, anti-desmoglein, IgG1, IgG4

## Abstract

Pemphigus vulgaris (PV) and pemphigus foliaceus (PF) are blistering disorders mediated predominantly by IgG1 and IgG4 antibodies directed towards desmoglein (Dsg)1 and/or 3. The anti-CD20 monoclonal antibody rituximab is a highly effective treatment for PV, although less is known regarding its efficacy in the treatment of PF and the dynamics of anti-Dsg1 IgG subclasses post rituximab treatment. We therefore investigated clinical outcomes and anti-Dsg antibody dynamics in 105 pemphigus patients (89 with PV, 16 with PF) treated with rituximab. Similar treatment responses were observed in PV and PF, although there were significant differences in anti-Dsg antibody dynamics post treatment, with a greater reduction of anti-Dsg1 in anti-Dsg1 positive PV than in PF which corresponded to a reduction in predominantly IgG4 subclass antibodies. Baseline clinical parameters that correlated with complete remission were increased age and lower disease duration. We identified lower circulating B cell counts in older patients and enrichment in anti-Dsg1 IgG4 and anti-Dsg3 IgA with longer disease duration, suggesting that immune senescence and increased clonal diversity could account for more and less favourable treatment responses respectively. Reductions in anti-Dsg1 post rituximab had greater prognostic value than anti-Dsg3 and this applied to both IgG1 and IgG4 subclasses. The study findings therefore suggest disparate disease biology underlying anti-Dsg1 and 3 responses and advances our knowledge of the biology underlying shorter disease duration and increased age as positive predictors of treatment response to rituximab.

## Introduction

Pemphigus vulgaris (PV) and pemphigus foliaceus (PF) are antibody mediated mucocutaneous blistering disorders. PV is characterised by mucosal and/or cutaneous ulceration due to the presence of anti-Dsg3 antibodies with or without anti-Dsg1 antibodies. PF is associated with anti-Dsg1 antibodies which cause superficial cutaneous blisters and erosions without mucosal involvement (1).

B cells play an important role in the pathogenesis of pemphigus by acting as precursors to antibody secreting cells. Pemphigus disease activity correlates with anti-Dsg1 and anti-Dsg3 antibody levels (2), therefore a goal of treatment is to reduce pathogenic antibody levels. The chimeric monoclonal anti-CD20 antibody rituximab has been demonstrated to be a highly effective treatment for PV and results in potent and prolonged peripheral B cell depletion with rates of complete or partial remission of 80-90% in PV patients (3–6). Factors that have been identified to predict better treatment response in PV include falls in both anti-Dsg1 and -Dsg3 antibodies (7–9), shorter disease duration (10), and higher baseline CD4 T cell counts prior to rituximab treatment (7). Fewer studies have documented the clinical efficacy of rituximab for PF versus PV, although a recent analysis of the RITUX 1 and 3 trials have demonstrated a lower relapse rate in PF patients (11). Furthermore, little is known about the dynamics of anti-Dsg 1 and 3 isotypes and IgG subclasses after rituximab and whether they correlate with clinical responses.

In this retrospective single centre study we investigate the efficacy of rituximab in PV and PF patients and investigate clinical and immunological parameters associated with clinical response.

## Methods

### Patients

Patients with PV and PF treated with rituximab between 2007 and 2021 were retrospectively identified by a systematic search of electronic health records. Eligibility criteria for PV and PF patients were as follows: (i) rituximab was to be administered to all patients as per the Rheumatoid Arthritis (RA) protocol as two 1-gram infusions, typically dosed 14 days apart, (ii) minimum patient follow-up period should have been 24 months and (iii) there was no prior treatment with rituximab. Exclusion criteria were as follows: (i) other subtypes of pemphigus that were not vulgaris or foliaceus; (ii) no baseline quantification of anti-Dsg antibodies or lymphocyte subsets; (iii) patients with no documented follow-up and (iv) incomplete dosing of rituximab.

### Data collection

Electronic patient records were used over a follow up period of 36 months to assess disease responses and changes in circulating B cell counts, T cell subsets (CD4+ and CD8+), natural killer (NK) cells and anti-Dsg1 and -Dsg3 antibody levels at <12 months, 12–24 months and >24 months post rituximab treatment. Anti-Dsg total IgG was quantified by our central diagnostic laboratory using anti-Dsg1 and anti-Dsg3 ELISA kits (MBL) according to the manufacturer’s instructions. Disease duration was defined as the time between symptom onset and the first dose of rituximab. Remission time was documented as the time between the first rituximab dose and the first disease flare. Peak rituximab response was categorised as complete remission (CR), partial remission (PR), or no response (NR), according to the internationally recognised 2008 consensus statements (12). PV patients were also subclassified according to the presence of mucosal, cutaneous or mucocutaneous lesions at baseline and by anti-Dsg 1 positivity pre-rituximab treatment, the cut off for positivity being >30U/ml. Given that this study sought to investigate outcomes following a single cycle of rituximab, if patients were retreated with rituximab during the follow up period, no further data regarding lymphocyte subsets or anti-Dsg antibody levels were collected.

### Lymphocyte subset quantification

Lymphocyte subsets were quantified from peripheral blood samples by our central diagnostic laboratory as part of clinically validated testing protocol according to ISO15189, using an AQUIOS CL Flow Cytometer System in combination with AQUIOS Tetra-1 and 2+ antibody panels. The tetra-1 panel consisted of CD45 fluorescein isothiocyanate (FITC), CD4 phycoerythrin (PE), CD8 electron coupled dye (ECD) and CD3 Phycoerythrin (PE)-Cyanine 5.5 (PC5). The Tetra-2+ panel consisted of CD45 FITC, CD56+CD16 PE, CD19 ECD and CD3 PC5. A representative gating strategy is displayed in **Supplementary Figure S1a**.

### Anti-desmoglein ELISA

Commercially available enzyme-linked immunosorbent assay (ELISA) kits manufactured by MBL Laboratories, Japan, were to detect IgG1 and IgG4 subclasses using goat anti-human IgG1 (Thermofisher) HRP secondary antibody at a dilution of 1:8000 and a mouse anti-human IgG4 (Invitrogen) HRP secondary antibody at a dilution of 1:30000. OD values for disease samples pre-rituximab treatment and negative controls are displayed in **Supplementary Figure S1b**. Antibody levels were expressed as Arbitrary Units that were calculated by comparison with a reference serum that had high levels of anti-Dsg1 or -Dsg3 antibodies. This same reference serum was run on each ELISA plate and Arbitrary Units were then calculated as: Sample Units = (sample OD/reference OD) *100. We developed an inhouse ELISA for the detection of anti-Dsg3 anti-IgM and IgA to allow a larger sample size to be investigated. ELISA plates were coated with recombinant Dsg3 (Kactus Ltd) at a concentration of 5ug/ml followed by blockade with 5% BSA. Serum was added at a concentration of 1:100 and secondary HRP anti-human IgM (Invitrogen) and IgA (Antibodies.com) were used at 1:7500 and 1:10000 dilutions respectively. To assess anti-Dsg3 positivity, a negative threshold of 0.35 and 0.3 for IgM and IgA was used respectively as indicated by background signal for anti-Dsg3 negative disease controls with PF (**Supplementary Figure S1c**).

### Statistical analysis

Graphs were made and statistical analysis was performed using GraphPad Prism version 10.1.1. Kaplan-Meier plots were created using GraphPad Prism and a log-rank (or Mantel-Cox) test was used to identify differences in survival between groups. Fisher’s exact test was utilized to identify differences in rates of CR, PR and NR in PF and PV patients. Tests involving [univariate and/or multivariate] logistic regression to identify predictors of treatment response were performed in R (version 2024.09.0) using the glm R package. A p-value of < 0.05 was deemed as statistically significant. Annotation of graphs to infer statistical significance were as follows; * = p<0.05, ** = p<0.01, *** = p<0.001, **** = p<0.0001, ns = no statistical significance.

## Results

### Rituximab is equally efficacious to treat both pemphigus foliaceus and vulgaris but results in disparate post-treatment anti-Dsg1 antibody dynamics

**Figure 1:**
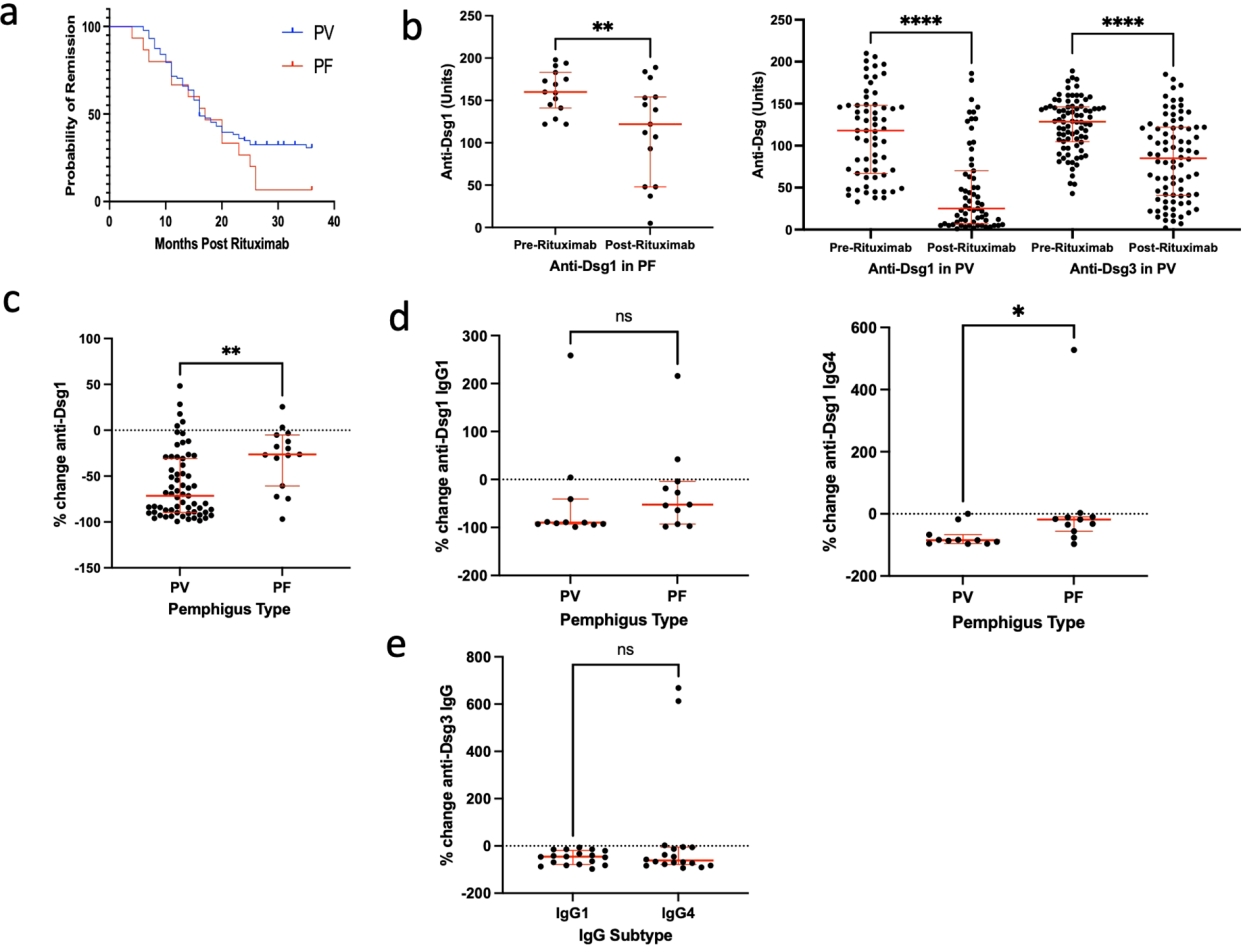
Rituximab is equally efficacious to treat both pemphigus foliaceus and vulgaris but results in disparate post-treatment anti-Dsg1 antibody dynamics. **(a)** Kaplan Meier curve demonstrating relapse rates in patients with PV and PF, p = 0.195 log-rank (or Mantel-Cox) test; **(b)** Dot plots depicting anti-Dsg1 and –Dsg3 antibody levels before and 6–12 months post rituximab treatment in patients with positive baseline levels (defined as >30U/ml), in patients with PF and PV. PF: n=15 (1 patient excluded as no post-rituximab Dsg1 level was measured), Anti-Dsg1 positive PV: n=63, anti-Dsg3 positive PV: n=82 (Wilcoxon signed-rank test); **(c)** Dot plot demonstrating percentage changes in anti-Dsg1 antibodies from baseline to 6–12 months post rituximab treatment in patients with PV and PF. Anti-Dsg1 positive PV: n=63, PF: n=15 (Mann Whitney test); **(d)** Dot plots demonstrating percentage changes in anti-Dsg1 IgG1 and IgG4 antibodies from baseline to 6–12 months post rituximab treatment in patients with PF and PV matched according to baseline anti-Dsg1 levels, n=11 (Mann Whitney test); **(e)** Dot plot demonstrating percentage changes in IgG1 and IgG4 anti-Dsg3 antibodies from baseline to 6–12 months post rituximab treatment, n=18 (Mann Whitney test). Error bars indicate the median value and interquartile range. * = p<0.05, ** = p<0.01, **** = p<0.0001. ns, no statistical significance.

Clinical responses of PF to rituximab and the dynamics of anti-Dsg1 antibody isotypes after treatment remain understudied. We therefore investigated remission rates and anti-Dsg antibody isotype levels in our cohort of 105 rituximab treated patients, 89 with PV and 16 with PF. Patient characteristics are presented in **Table 1**. Overall, 65% of pemphigus patients achieved CR, 23% achieved PR and 12% had NR and there were no significant differences in treatment response or duration of remission in patients with PF versus PV (**Figure 1a**, **Supplementary Figure S1d**). Baseline and adjuvant treatments continued after rituximab are listed in **Supplementary Table S1** and did not impact on treatment responses. By analysing data from our central diagnostic lab that measures total anti-Dsg1 and -Dsg3 IgG levels, a greater reduction in anti-Dsg1 antibodies was observed in patients with anti-Dsg1 positive PV versus PF (**Figures 1b, c**). We next investigated anti-Dsg1 IgG1 and IgG4 subclass levels by modulating a commercially available ELISA system (MBL), as these are known to represent the most abundant subclasses in pemphigus (13). Serum samples from 12 PF patients were available for analysis and were matched with samples from patients with anti-Dsg1 positive PV with similar (+/-10%) baseline anti-Dsg1 levels. Baseline anti-Dsg1 IgG1 and IgG4 levels were similar in PV versus PF (**Supplementary Figure S1e**). Both IgG1 and IgG4 anti-Dsg1 antibodies fell post rituximab, but a greater reduction of anti-Dsg1 IgG4 was seen in PV compared to PF patients, whilst there was no difference in the degree of IgG1 reduction (**Figure 1d**). There was no difference in the reduction of IgG1 and IgG4 anti-Dsg3 antibodies in PV patients (**Figure 1e**).

**Table 1:** Patient demographics and clinical data.

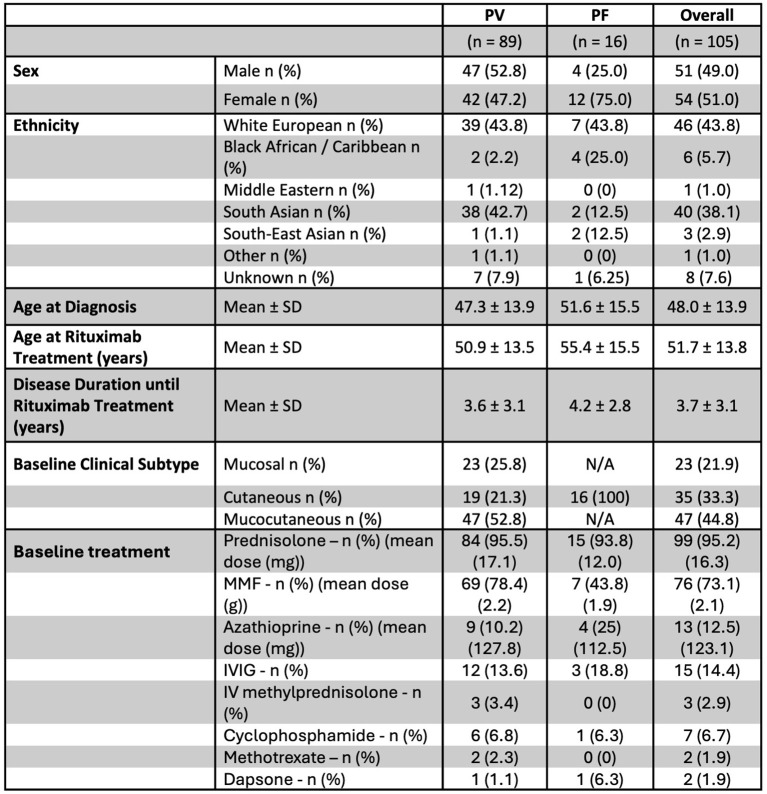

Key: n, number of patients; %, proportion of all patients; SD, standard deviation; IVIG, intravenous immunoglobulin; MMF, mycophenolate mofetil; mg, milligrams; g, grams.

Our data therefore demonstrate equal clinical responses to rituximab in both PV and PF patients but significant differences in the serological response. The anti-Dsg1 reduction in anti-Dsg1 positive PV was predominantly due to a reduction of IgG4 subclass antibodies, which demonstrate that they represent the most rituximab responsive subclass. The lower reduction in anti-Dsg1 in PF but equal response to rituximab is intriguing and suggests disparate disease biology from PV.

### Baseline clinical and serological predictors of response to rituximab

**Figure 2:**
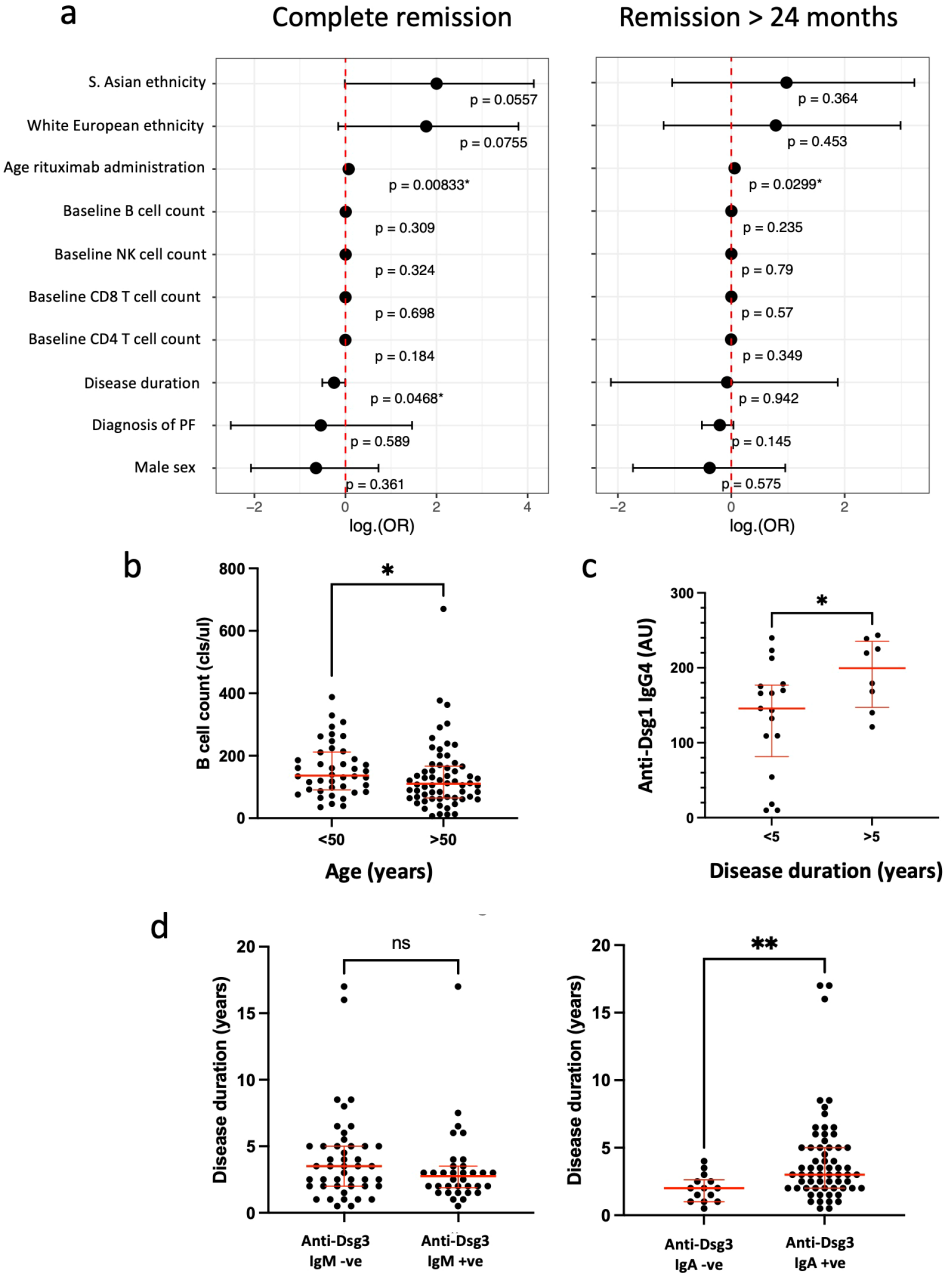
Baseline clinical and serological predictors of response to rituximab. **(a)** Forest plot showing the adjusted odds ratios for response to rituximab, with the endpoints of complete remission (left) and remission greater than 24 months (right). The logistic regression model was adjusted for patient demographics (age, sex, race/ethnicity), disease characteristics (duration, PV/PF diagnosis), and serological predictors (baseline B-cell, NK-cell, CD8+ T-cell, CD4+ T-cell). Error bars represent the limits of the 95% confidence interval for the odds ratio. *P* values are shown for each predictor. The red dashed line indicates an odds ratio of zero; **(b)** Dot plot demonstrating baseline B cell counts in patients <50 (n=42) and >50 years old at rituximab administration (n=62) (Mann Whitney test). 1 patient was excluded as no baseline B cell count available; **(c)** Dot plot demonstrating anti-Dsg1 IgG4 levels in patients with PF and anti-Dsg1 positive PV with disease duration <5 (n=17) and >5 years (n=8) (Mann Whitney test); **(d)** Dot plots demonstrating disease duration in patients with positive and negative anti-Dsg3 IgM and IgA antibodies (Mann Whitney test). Anti-Dsg3 IgM -ve: n=45, Anti-Dsg3 IgM +ve: n=34, Anti-Dsg3 IgA -ve: n=14, Anti-Dsg3 IgA +ve: n=63. Error bars indicate the median value and interquartile range. * = p<0.05, ** = p<0.01. ns, no statistical significance.

We next investigated factors that could predict rituximab treatment response and duration in our cohort of PV and PF patients. Using logistic regression run on baseline parameters on all patients, in accordance with previous studies we observed that greater age was associated with CR and prolonged remission, whilst greater disease duration was associated with lower rates of CR (**Figure 2a**, **Supplementary Figure S2a**) (14). A sub analysis of our PV patient cohort also demonstrated significant correlation between age of rituximab administration and additionally South Asian ancestry with complete remission but not prolonged remission (**Supplementary Figure S2b**). We next investigated baseline immunological parameters that correlate with age. Patients over the age of 50 had lower baseline B cell counts than those under the age of 50 (**Figure 2b**), but the frequencies of CD4 T cells, CD8 T cells and NK cells were not different (**Supplementary Figure S2c**). Patients with disease duration of more than 5 years also had higher levels of IgG4 anti-Dsg1 antibodies but not anti-Dsg-1 IgG1, anti-Dsg3 IgG1 or IgG4 antibodies (**Figure 2c**, **Supplementary Figure S2d**). We next investigated IgM and IgA anti-Dsg3 antibodies in PV. Serum was available from 79 patients for analysis and 43% and 82% of patients were positive for anti-Dsg3 IgM and IgA respectively (**Supplementary Figure S1c**). Disease duration was higher in patients with anti-Dsg3 IgA antibodies but not IgM antibodies (**Figure 2d**). This was not due to increased age as disease onset was not later in patients with or without IgM and IgA anti-Dsg3 antibodies (**Supplementary Figure S2e**). We next performed a logistic regression analysis to investigate whether baseline anti-Dsg1 and -3 IgG and anti-Dsg3 IgM or IgA predicted complete response or duration of response greater than 24 months. We identified no correlation between pre-treatment antibody levels and complete or prolonged remission (**Supplementary Figure S3a**).

In summary, increased age at rituximab administration was associated with lower circulating B cell frequencies at baseline and higher rates of complete remission post-treatment. Conversely, increased disease duration was associated with lower rates of complete remission but higher levels of IgG4 anti-Dsg1 and IgA-anti-Dsg3 antibodies which was suggestive of greater clonal diversity.

### Clinical correlation of anti-desmoglein antibody dynamics and response to rituximab

**Figure 3:**
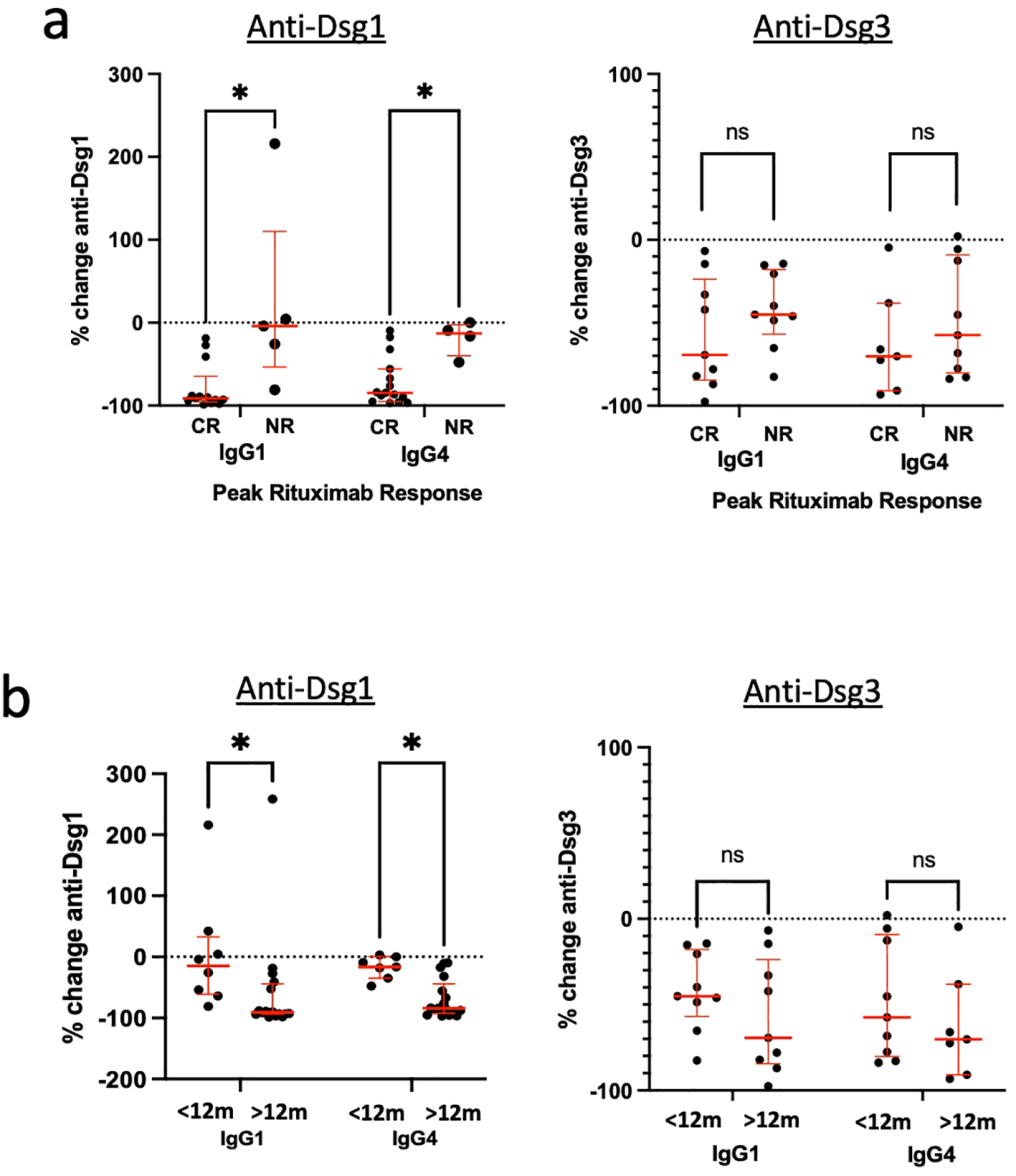
Clinical correlation of Desmoglein antibody dynamics post rituximab. **(a)** Dot plots demonstrating percentage change in anti-Dsg1 and -Dsg3 IgG1 and IgG4 antibody levels from baseline to 6–12 months after treatment in patients with CR and NR. Anti-Dsg1 IgG1 CR: n=13, NR: n=5. Anti-Dsg1 IgG4 CR: n=15, NR: n=4. Anti-Dsg3 IgG1 CR: n=9, NR: n=9. Anti-Dsg3 IgG4 CR: n=7, NR: n=9 (Mann Whitney test); **(b)** Dot plots demonstrating percentage change in anti-Dsg1 and 3 IgG1 and IgG4 antibody levels from baseline to 6–12 months after treatment in patients with remission <12 months and >12 months. Anti-Dsg1 IgG1 <12m: n=8, >12m: n=16. Anti-Dsg1 IgG4 <12m: n=7, >12m: n=17. Anti-Dsg3 IgG1 <12m: n=9, >12m: n=9. Anti-Dsg3 IgG4 <12m: n=9, >12m: n=7 (Mann Whitney test). Error bars indicate the median value and interquartile range. * = p<0.05. ns, no statistical significance.

Given that baseline anti-Dsg3 antibodies did not correlate with clinical response to rituximab, we next investigated the changes in anti-Dsg antibodies pre- and 6–12 months post rituximab treatment. In accordance with previous studies, we observed greater reduction in anti-Dsg1 than anti-Dsg3 antibodies in PV patients who achieved CR versus NR (**Supplementary Figure 3b**) (14). Falls in both IgG1 and IgG4 anti-Dsg1 antibodies were greater in patients achieving CR versus NR, but there was no difference in the fall of IgG1 and IgG4 anti-Dsg3 antibodies (**Figure 3a**). Early relapse (<12 months) was associated with smaller reductions in anti-Dsg1 IgG1 and IgG4 levels, but not anti-Dsg3 IgG1 and IgG4 levels post-rituximab treatment (**Figure 3b**).

These data therefore support the equal predictive value of both IgG1 and IgG4 anti-Dsg 1 antibodies rituximab in predicting higher rates of CR and prolonged remission.

## Discussion

In this large single centre study we investigated treatment responses and anti-Dsg antibody dynamics in patients with PF and PV that were treated with rituximab. We demonstrate a 65% CR rate of all pemphigus patients following a single cycle of rituximab over a 24 month follow up period. Our treatment outcomes are in concordance with the existing literature, with studies reporting CR rates ranging from 42% to 79% after a single cycle of rituximab (3–5, 15). These response rates are, however, lower than sequential rituximab dosing which can result in remission rates off adjuvant therapy of over 90% (11, 16). Treatment responses of PF to rituximab are relatively understudied compared to PV and our data confirms equal efficacy in both diseases.

Despite observing similar clinical responses to rituximab in PF and PV patients we observed different dynamics in anti-Dsg1 antibodies. Reduction in anti-Dsg1 IgG levels post rituximab is known to be a better predictor of remission in PV than anti-Dsg3 level reduction (14), but the greater reduction in anti-Dsg1 levels we observed in anti-Dsg1 positive PV is intriguing and suggests different disease biology underlying anti-Dsg1 and anti-Dsg3 responses. Furthermore, we identified that anti-Dsg1 positive PV patients had greater reductions in anti-Dsg1 than PF patients, and that this represented predominantly IgG4 subclass antibodies. This therefore highlights that anti-Dsg1 IgG4 antibodies represent the most rituximab responsive subclass and their greater reduction in PV vs PF may suggest disparate disease biology. The biological basis for these observations warrants further investigation, but may relate to antibody secreting cell (ASC) survival. ASCs are thought to be short lived and due to their lack of CD20 expression are not directly targeted by rituximab. The resulting fall in anti-Dsg antibodies post rituximab treatment is therefore postulated to be due to the demise of short lived ASCs and lack of B cell progenitors to renew them (17). The differential anti-Dsg antibody dynamics we have observed in PV versus PF may therefore suggest Dsg-1 and Dsg-3 ASC have different longevity, and that those of IgG4 subclass may have a shorter longevity than the IgG1 subclass. Further work is, however, needed to address this critical element of human immunology which is central to the persistence and treatment of antibody driven autoimmune diseases.

In concordance with other studies we confirm greater age at disease onset and rituximab administration is associated with more favourable treatment responses (14). Of interest, this association has also been observed in myasthenia gravis which like pemphigus is an IgG4 predominant autoimmune disease, but not rheumatoid arthritis (18, 19). The reasons underlying this phenomenon are not understood but our observation of lower circulating B cell counts in older patients suggests that immune senescence may play a role. In keeping with this, Saha et al. (20) found that older patients had overall shorter pemphigus disease durations. However, T cell subsets were not altered with advanced age and in contrast to previous studies we did not find that CD4 T cell frequencies predicted treatment outcomes (7). Longer disease duration is also known to negatively impact on rituximab response (21). We demonstrate that longer disease duration is associated with higher levels of IgG4 anti-Dsg1 and anti-Dsg3 IgA antibodies. This may represent increased clonal diversity as a result of prolonged disease which may negatively impact on rituximab treatment outcomes. In keeping with this, Golinski et al. identified that the presence of three or more anti-Dsg3 IgG subclasses predicted relapse (13).

CR versus NR post-rituximab treatment was associated with equal falls in anti-Dsg1 IgG1 and IgG4 levels but not anti-Dsg3 IgG1 and IgG4 levels. The predominance and pathogenicity of anti-Dsg 1 IgG4 levels has been documented in numerous studies of endemic pemphigus, PF and PV (22, 23). Our finding that PV patients who lack response to rituximab fail to reduce both IgG1 and IgG4 anti-Dsg1 antibodies further supports the pathogenicity of both subclasses and the greater prognostic value of a fall in anti-Dsg1 versus anti-Dsg3 antibodies.

In summary, this study advances our understanding of anti-Dsg1 and 3 dynamics post rituximab treatment. It identifies intriguing differences in anti-Dsg1 levels between PF and PV that suggests disease associated differences that warrant further investigation. This study also adds to the data surrounding the efficacy of rituximab as a treatment for PF. Finally, it identifies serological and immunological factors that associate with increased age and shorter disease duration which may account for their association with improved treatment outcomes.

## Data Availability

The raw data supporting the conclusions of this article will be made available by the authors, without undue reservation.
