# Supplementary figures and images for: Clinical correlation of anti-desmoglein antibody dynamics in pemphigus treated with rituximab
Source: Front Immunol. 2025 Dec 12;16:1713987. doi: 10.3389/fimmu.2025.1713987 (PMC12741074; doi:10.3389/fimmu.2025.1713987)

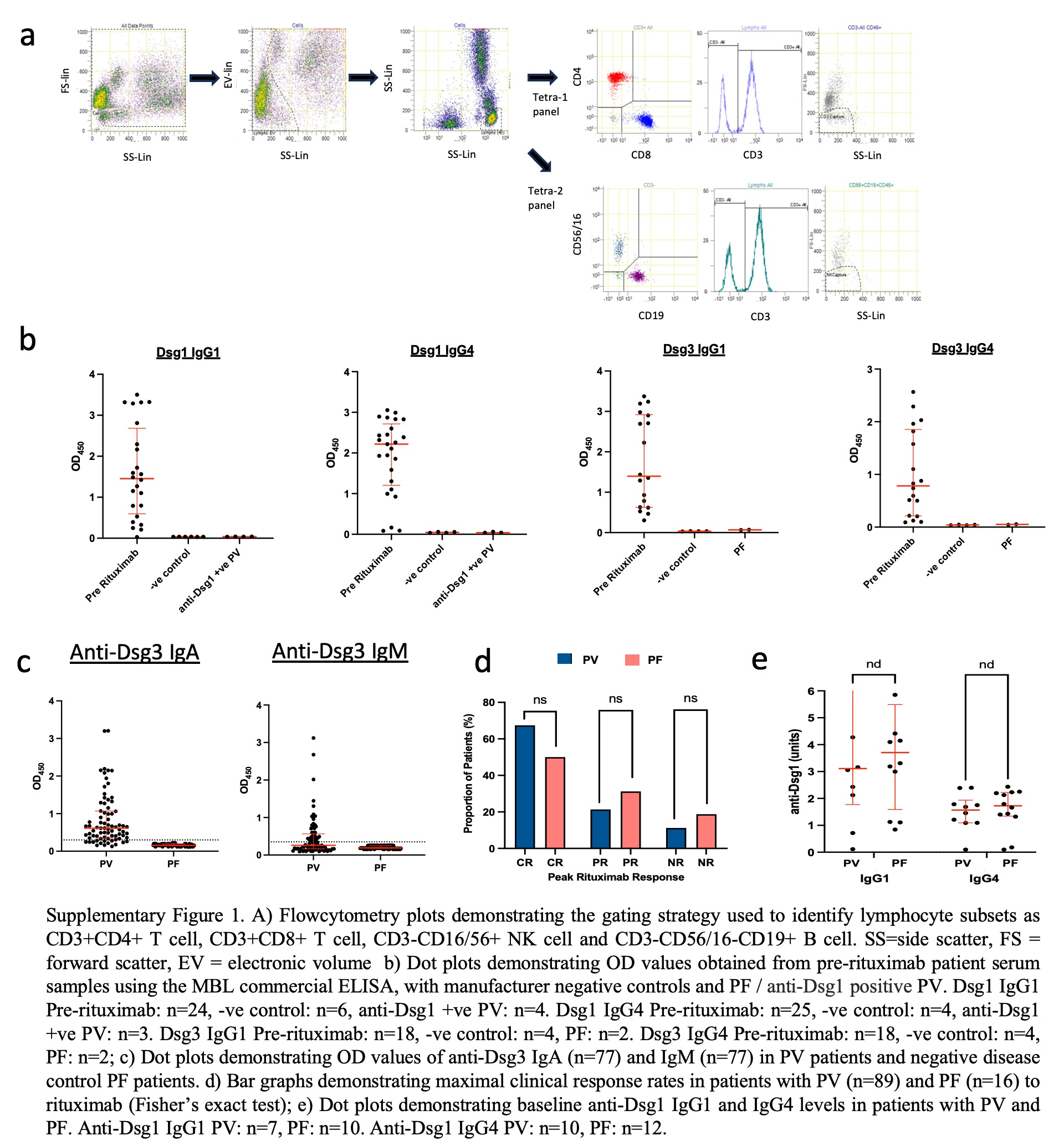

Supplement: Supplementary file 1 [file Image1.jpg]

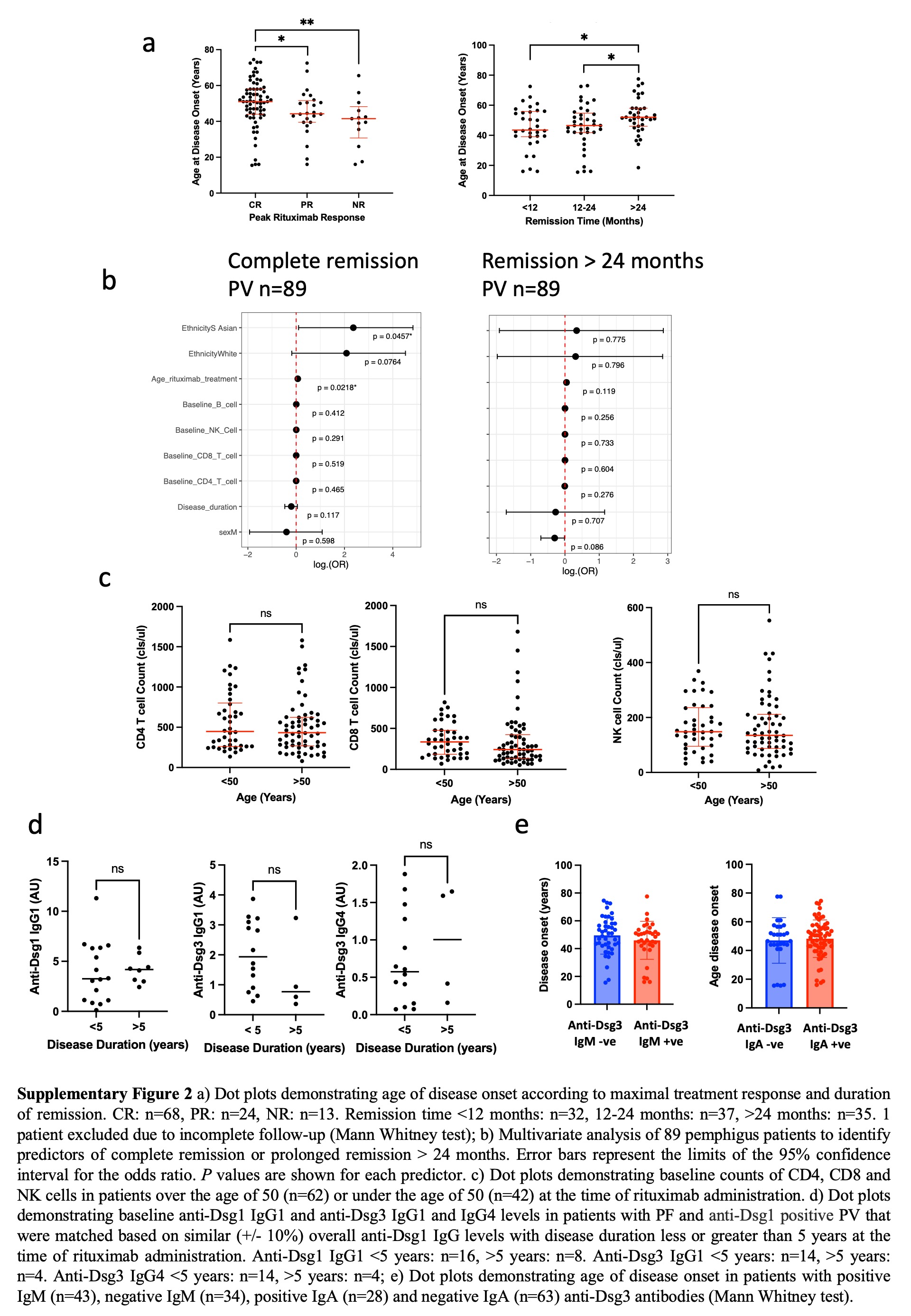

Supplement: Supplementary file 2 [file Image2.jpg]

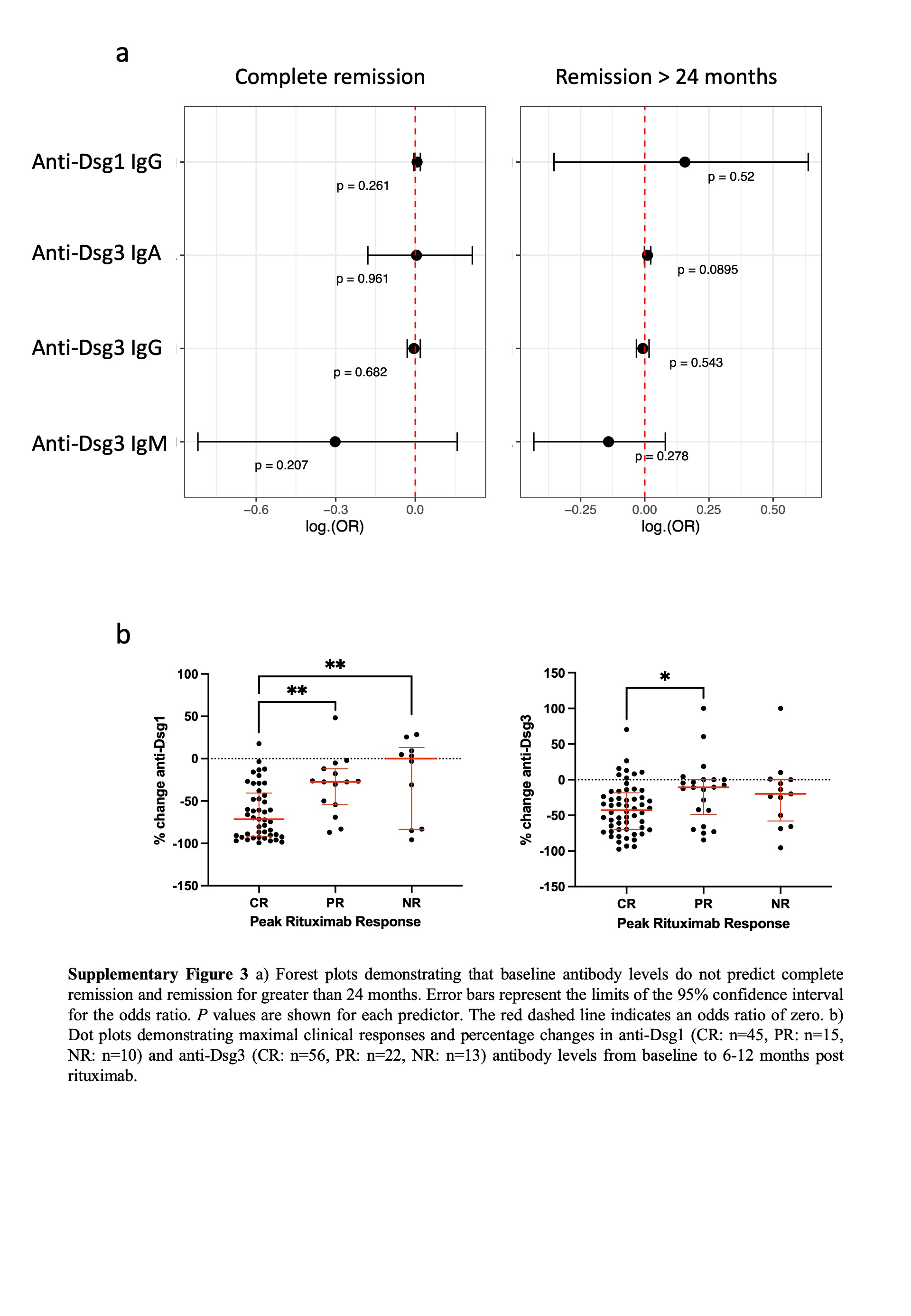

Supplement: Supplementary file 3 [file Image3.jpg]

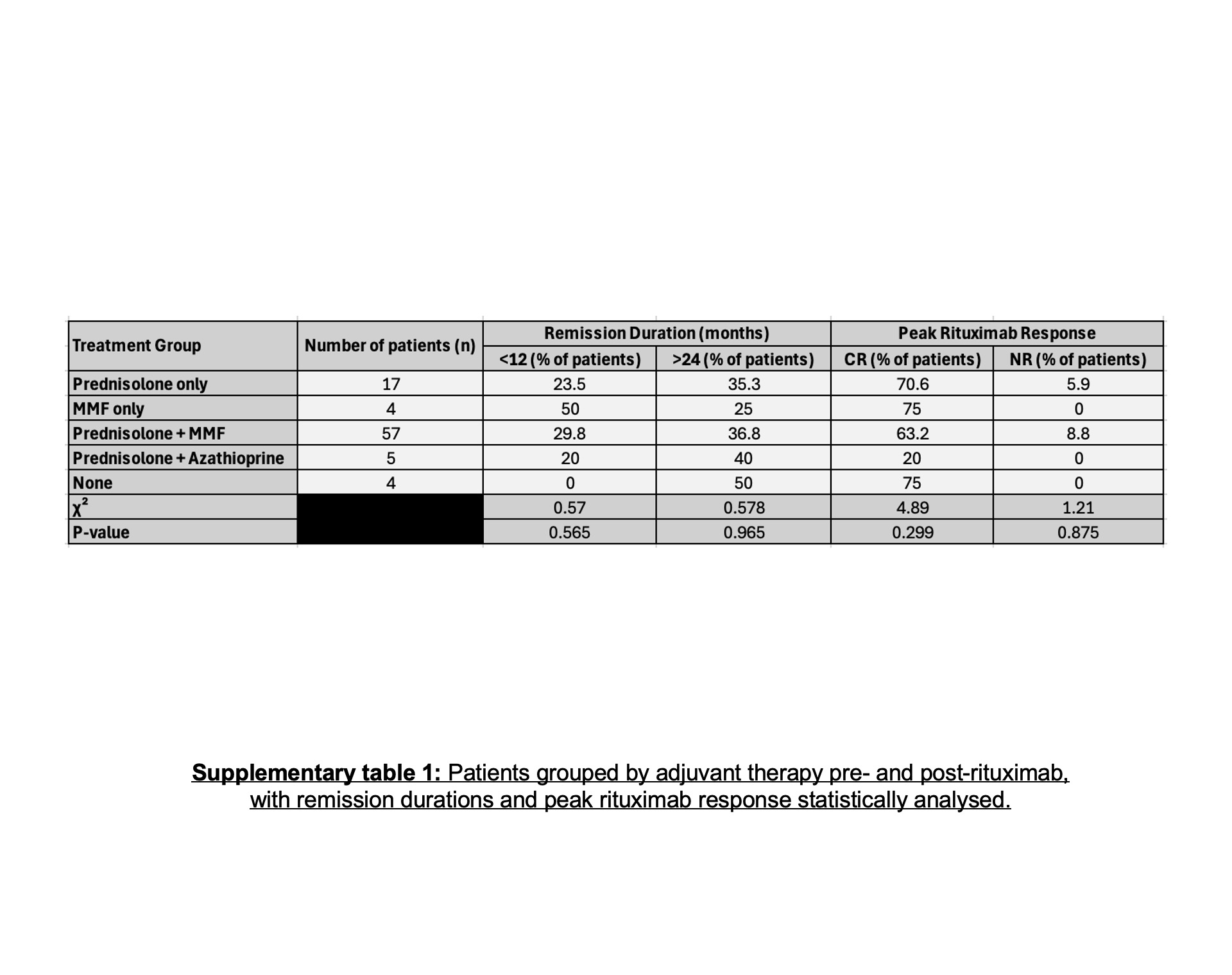

Supplement: Supplementary file 4 [file Image4.jpeg]
